# Vacuum-Induced Surface Freezing to Produce Monoliths of Aligned Porous Alumina

**DOI:** 10.3390/ma9120983

**Published:** 2016-12-05

**Authors:** Sandra Großberger, Tobias Fey, Geoffrey Lee

**Affiliations:** 1 Division of Pharmaceutics, University of Erlangen, Cauerstrasse 4, 91054 Erlangen, Germany; sandra.großberger@fau.de; 2 Department of Material Science & Engineering, University of Erlangen, Martenstrasse 5, 91058 Erlangen, Germany; tobias.fey@fau.de

**Keywords:** directional freezing, surface freezing, vacuum, aligned pores, alumina, sinter

## Abstract

Vacuum-induced surface freezing has been used to produce uni-directional freezing of colloidal aluminum oxide dispersions. It leads to zones of different structure within the resulting sintered monoliths that are highly similar to those known for freeze casting using a cryogen cold source. A more-or-less dense surface layer and a cellular sub-surface region are formed, beneath which is a middle region of aligned lamellae and pores that stretches through most of the depth of the monolith. This is the case even at a volume fraction of dispersed phase as low as 0.032. A more-dense but still porous base layer is formed by accumulation of rejected nanoparticles preceding the freezing front and differs from previous reports in that no ice lenses are observed. X-ray micro-computed tomography reveals a uniform aligned pore structure vertically through the monolith. The pores close to the periphery are oriented radially or as chords, while the center region contains domains of parallel pores/lamellae. The domains are randomly oriented to one another, as already reported for regular freeze casting. This technique for directional freezing is convenient and easy to perform, but requires further refinement in that the temperature gradient and freezing rates remain yet to be measured. Also, control of the temperature gradient by varying chamber vacuum and shelf temperature needs to be evaluated.

## 1. Introduction

The principles of freeze casting to prepare aligned porous materials date back to the 1960s and can be applied to colloidal dispersions of ceramics [1,2,3] and polymers [4] as well as metals [5]. It involves the directional freezing of the colloidal dispersion of the material, which allows the growth of elongated, unidirectionally-aligned ice crystals that have very high aspect ratios [6]. This elongated shape is caused by the strongly anisotropic growth kinetics of crystals of hexagonal ice, I_h_ [7], perpendicular to the surface of the cold-source [6]. As they grow, the chimney-like ice crystals expel the colloidal particles present in the original dispersion to the boundaries between adjacent ice crystals. This expulsion is caused by the predominance of the repulsive forces acting between the colloidal particles and the growing ice crystal surface over the drag force hindering their motion. The minimal ice growth velocity that is necessary to expel a particular particle size is readily calculated [8]. The expelled particles aggregate and form thin lamellae between the ice crystals [9]. Subsequent lyophilization removes the ice and leaves behind aligned, elongated channels, i.e., the ice “ghosts”, whose thin walls (“lamellae”) are composed of the aggregated colloidal particles [7]. The architecture of these pores can be varied by the use of different solvents such as water, camphene [10] and tertiary butyl alcohol [11], or additives such as glycerol [12], isopropanol [13] and zirconium acetate [14], as well as by the freezing rate [7] and the slurry concentration [15]. If a ceramic is used, the lyophilized green body can be sintered to produce a stable, highly-porous material that is composed of numerous parallel rows of elongated channels (ice ghosts) separated by thin lamellae of the ceramic material [3].

The routine method of freeze casting is done by directional freezing of a ceramic slurry contained in a mould placed on a cold finger [16] in contact with a cryogen [8], followed by transfer to a lyophilizer for sublimation and then to an oven for sintering. We have attempted to simplify this technique by introducing vacuum-induced surface freezing to produce directional ice crystal growth. Vacuum-induced surface freezing was originally developed by us to freeze aqueous carbohydrate solutions as part of an accelerated lyophilization process [17]. The chamber pressure was reduced such that the surface of the solution froze to a thin layer. On release of the vacuum and reduction of shelf temperature, a directional ice crystal growth then occurred to produce elongated, chimney-like ice crystals. The gross morphology of the lyophilized carbohydrate [17] appears similar to the structures produced by conventional freeze casting of ceramics [3,4,6,7]. The technique is easy to apply, requires no complex freezing set-up, and is performed from start to finish in the drying chamber of the lyophilizer. In our work we introduce a simplification of the original technique of vacuum-induced surface freezing [17] in that the vacuum is maintained (and not released) after incipient surface freezing until the complete sample has directionally frozen through. One difference between this technique and conventional freeze casting that might influence product morphology is that the degree of supercooling and hence freezing rate may be different [18], although this has never been measured. It remains therefore to be seen if the pore morphology is that of the three zone-pattern of dense layer, cellular layer and lamellar layer described for liquid nitrogen-driven cold-finger freezing of ceramic dispersions [16]. We describe in this paper our efforts to use vacuum-induced surface freezing of colloidal aluminum oxide dispersions to produce monoliths having elongated, aligned pores. The structure of these monoliths was examined using a combination of scanning electron microscopy and micro-computed tomography. The aim was to demonstrate if these aligned structures produced by vacuum-induced surface freezing were the same as those known for regular freeze casting.

## 2. Results and Discussion

**Figure 1:**
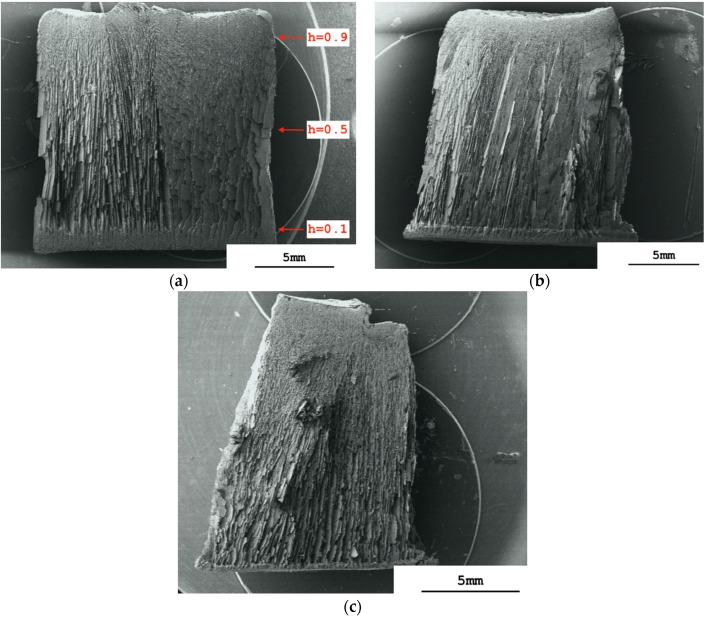
Scanning electron micrographs of longitudinal sectioned sintered monoliths prepared from alumina slurries having different volume fractions of dispersed phase, φ_v_. (**a**) φ_v_ = 0.128; (**b**) φ_v_ = 0.064; (**c**) φ_v_ = 0.032. Note that the red arrows demark the planes of the cross-sections examined later by µ-CT; h = positional height within monolith (0 ≤ h ≤ 1).

The sintering shrinkage of each of the monoliths was anisotropic, being approximately 17% in the vertical direction and 25% in the horizontal direction. The scanning electron micrograph of a longitudinal section cut through a sintered monolith prepared from an alumina slurry having a volume fraction of dispersed phase, φ_v_, of 0.128 (=50% *w*/*w* alumina) shows a vertically aligned structure, which starts at the monolith’s top surface and reaches down through almost its complete depth (Figure 1a). It comprises parallel layers of lamellae and pores. The slurry surface freezes as the chamber vacuum reaches and passes through approximately 450 mTorr (≈0.6 mbar) while the shelf temperature, T_shelf_, is still at +10 °C. This is caused by evaporative water loss from the slurry surface at low pressure, where the endothermic enthalpy of vaporization causes sufficient cooling of the slurry to produce freezing of its surface [17,19]. The frozen surface layer is colder than the vial base and directional ice crystal growth is initiated perpendicular to the location of the cold-source [6], i.e., from top to bottom through the glass vial containing the slurry. We found that it was not necessary to release the vacuum after incipient surface freezing to prevent boiling, as had been reported before [17]. It follows that endothermic cooling had reduced the surface temperature to below that of the triple point. The final vacuum of 150 mTorr (≈0.2 mbar) could be maintained during the complete process of directional freezing, it only being necessary to reduce the shelf temperature from +10 °C to −18 °C once the complete sample was frozen through to prevent melting. The aligned structure visible in Figure 1a stretches from the top surface down almost to the base of the monolith. Only in the lowest region close to the base is a change in morphology evident, where a more dense layer of thicker lamellae is seen.

Figure 1a also shows that the orientation of the vertically-aligned layers of lamellae and pores across the longitudinal section is strong, but not completely uniform. The left-hand region of the monolith has been sectioned at approximately a right-angle to the vertical run of the lamellae, whereas the right-hand region has been sectioned more obtusely. This same non-uniform pattern of orientation of the pores and lamellae is also observed on reducing the slurry concentration to φ_v_ = 0.064 in Figure 1b and, to a less obvious extent, down to φ_v_ = 0.032 in Figure 1c. We deduce that the lamellae and pores sectioned at a right-angle exist as parallel chords running across the diameter of the monolith, whereas the obtusely-sectioned lamellae and pores run radially from the circumference inwards. The radial orientation suggests that some ice crystal growth also occurs from the vial wall inwards through to the center of the monolith. The vial wall would also now be acting as a cold source, i.e., is colder than the center longitudinal axis of the vial, as is the case with regular vial lyophilization [20]. There are then both horizontal and lateral temperature gradients across the sample at some time during freezing. The lateral gradient may be present for a short time after incipient surface freezing, but the vertical gradient must surely predominate to give the strongly vertical orientation of the lamellae and pores observed in Figure 1a–c. Ice crystal growth is predominantly in the vertical direction when using vacuum-induced surface directional freezing. It is therefore the same as that observed with regular freeze casting of ceramic dispersions using, for example, a liquid nitrogen-driven cold-finger [21].

**Figure 2:**
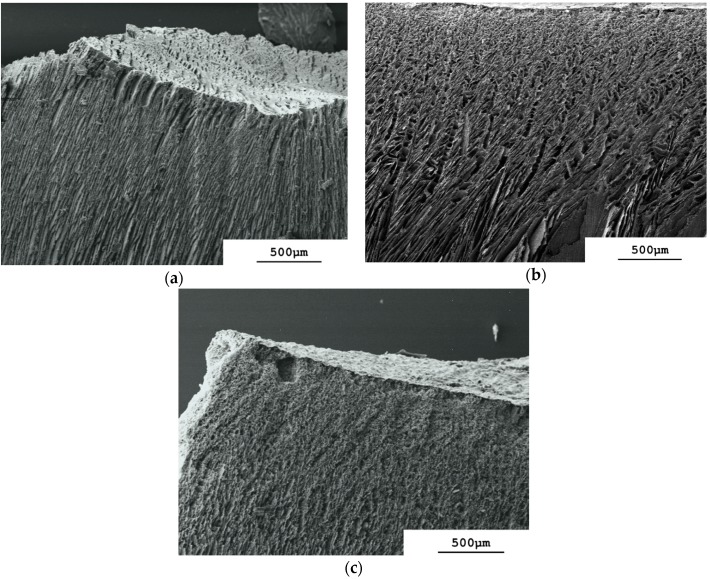
Scanning electron micrographs of longitudinal sectioned sintered monoliths to show surface and sub-surface structures. (**a**) φ_v_ = 0.128; (**b**) φ_v_ = 0.064; (**c**) φ_v_ = 0.032.

The longitudinal sections in Figure 1a–c show that zones of different appearance can be distinguished: a more-or-less dense top surface “skin”; a non-aligned porous sub-surface region; an aligned middle region; and a dense but still porous base region. The upper three correspond visually to the dense, cellular and lamellar regions reported for regular freeze casting using a liquid nitrogen-driven cold-finger for freezing of ceramic dispersions [16]. The zones formed differ with slurry concentration. With the most concentrated slurry, i.e., φ_v_ = 0.128, the sintered monolith’s top surface shows no “skin” and clearly the presence of pore openings (Figure 2a). At the lower slurry concentrations, φ_v_ = 0.064 (Figure 2b) and 0.032 (Figure 2c), the monoliths’ top surfaces appear, however, as a dense, skin-like, non-porous layer. Differences in structure are also observed in the sub-surface region immediately under the top surface at the different slurry concentrations. With φ_v_ = 0.128 the sub-surface region is made up of aligned pores that extend down from the monolith's surface (Figure 2a). This means that vacuum-induced surface freezing produces aligned ice crystals in this sub-surface layer that is directly in contact with the cold source, i.e., the frozen surface. This picture is changed at the lower slurry concentrations. With φ_v_ = 0.064 the immediate sub-surface region has rather a granular-appearance indicating a cellular porosity (Figure 2b). An aligned pore structure is visible only deeper into the sintered monolith. At φ_v_ = 0.032 (Figure 2c) the granular sub-surface region with cellular porosity extends even deeper before aligned pores are seen. These granular sub-surface regions with φ_v_ = 0.064 and 0.032 have the same appearance as the cellular transition zone found between the dense and lamellar zones produced when using a liquid nitrogen-driven cold-finger [16,21]. The extent (depth) of this cellular region is inversely proportional to the slurry concentration; at φ_v_ = 0.128 there is no cellular zone but rather aligned lamellar pores; at φ_v_ = 0.064 the cellular zone is 300 µm thick, and at φ_v_ = 0.032 it is >500 µm thick.

The morphology of the surface skin and the sub-surface region can be explained by the effects of slurry concentration—i.e., φ_v_—on the degree of supercooling of the slurry that occurs during vacuum-induced surface freezing. Deville et al. [22] found that formation of cellular or lamellar ice crystal morphology in alumina slurries frozen in a liquid nitrogen-driven cold-finger is a question of the degree of supercooling that takes place in the sample. A high degree of supercooling produces a large freezing front velocity and a cellular architecture, and above a critical velocity leads to particle engulfment and a dense non-porous structure [16]. A lower degree of supercooling reduces the freezing front velocity and allows formation of a lamellar structure. These authors also reported that the degree of supercooling is influenced strongly by the size of the nanoparticles forming the dispersed phase [23]. The larger the nanoparticles are, the smaller is their specific surface area and the lower is the number of nucleation sites located on the nanoparticle surface. Increasing the nanoparticle size will therefore result in a higher degree of supercooling of the freezing slurry, which will favor formation of a cellular or even dense-zone architecture. In our work the nanoparticle size was not altered, but rather the slurry concentration was varied. The highest concentration of φ_v_ = 0.128 in Figure 2a will have the largest total surface area of dispersed phase and the largest number of nucleation sites. The degree of supercooling reached on vacuum-induced surface freezing will therefore be low. Consequently the freezing front velocity moving down through the surface region is low and a porous surface with underlying lamellar architecture is formed. Neither a dense-zone nor a cellular region is observed in Figure 2a. Reducing the slurry concentration to φ_v_ = 0.064 (Figure 2b) and then further to 0.032 (Figure 2c) results in less nucleation sites, higher degrees of supercooling, and the formation of both a dense surface skin by particle engulfment and an underlying region of cellular morphology.

This is therefore just the behavior reported for zirconia-toughened alumina slurries frozen by regular freeze casting using a liquid nitrogen-driven cold-finger [24] where increasing φ_v_ took place, and hence a larger number of nucleation sites caused less supercooling before the onset of ice crystallization. At φ_v_ = 0.15 (cf. φ_v_ = 0.128 in Figure 2a) only a very thin dense layer was reported to be formed for a temperature gradient across the sample of 25 °C and a controlled cooling rate of 1 °C·min^−1^. Vacuum-induced surface freezing shows therefore the same influence of slurry concentration as seen with the liquid nitrogen-driven cold-finger. We note that the temperature gradient and freezing rate obtained with the former technique are as yet unknown. The rate of advancement of the freezing front through the monolith can, however, be estimated from published photographs of the vacuum-induced freezing process for φ_v_ = 0.128 as being initially approximately 30 µm/s [18]. This value is at first sight less than the critical velocity of >100 µm/s calculated for engulfment of sub-micrometer particles [21]. This critical velocity was calculated using a model for engulfment of an isolated single nanoparticle by a moving ice surface [8] and is likely to be lower when inter-particulate interactions operate in a concentrated slurry, as examined here.

**Figure 3:**
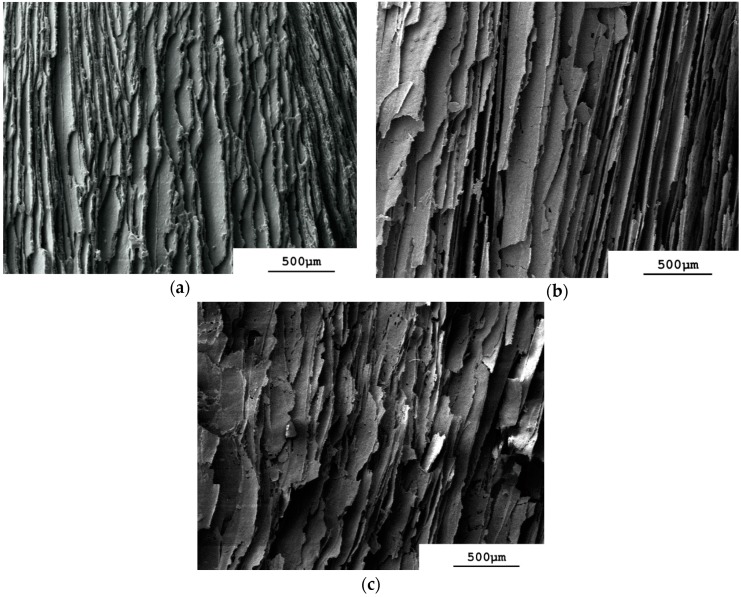
Scanning electron micrographs of longitudinal sectioned sintered monoliths to show central region of aligned lamellar pores. (**a**) φ_v_ = 0.128; (**b**) φ_v_ = 0.064; (**c**) φ_v_ = 0.032.

The parallel arrangement of aligned lamellae and pores that is present through most of the depth of the monolith prepared from the φ_v_ = 0.128 slurry (Figure 3a) is formed by directional freezing from the frozen surface layer downwards. It is similar to that reported for freeze-cast ceramics prepared from the liquid nitrogen-driven cold-finger technique [13,21,25]. An aligned pore morphology comes from an approximately steady-state velocity of the freezing-front that produces continuous ice crystals of constant thickness running through a dispersion [16,21]. The aligned lamellae in Figure 3a appear dense and without holes. Reducing the slurry concentration to φ_v_ = 0.064 keeps the same aligned lamellae and pore structure in this central region of the monolith (Figure 3b) but the lamellae appear to be thinner and less dense and show cracks. This is more pronounced with φ_v_ = 0.032 (Figure 3c), where the lamellae show numerous perforations. Note that we did no SEM image analysis to measure the lamellae thickness because this was performed later with µCT (see Figure 7, to be discussed presently). Lowering the slurry concentration has already been shown to increase the porosity of regularly freeze-cast structures [15] and hence also their structural wavelength, λ, equal to the repeat distance of the aligned structure [24]. The use of vacuum-induced surface freezing produces therefore the same aligned structure of lamellae and pores seen with regular freeze casting using a liquid nitrogen-driven cold-finger. This extends through most of the depth of the monolith, even at slurry concentrations as low as φ_v_ = 0.032 = 12.5% *w*/*w*.

**Figure 4:**
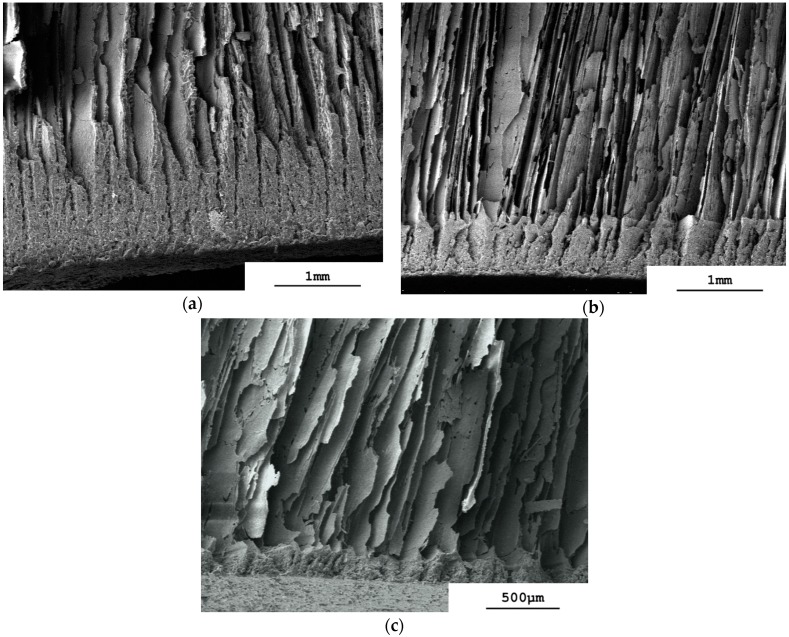
Scanning electron micrographs of longitudinal sectioned sintered monoliths to show detail of base region showing dense appearance. (**a**) φ_v_ = 0.128; (**b**) φ_v_ = 0.064; (**c**) φ_v_ = 0.032.

The aligned structure is altered in the base region of the sintered monoliths at all values of φ_v_. The aligned pores and lamellae are still identifiable almost right down to the vial base, but then transform to a more dense structure with low porosity close to the vial base. This base layer is thickest at φ_v_ = 0.128 (Figure 4a) and becomes thinner as the slurry concentration is reduced through φ_v_ = 0.064 (Figure 4b) to φ_v_ = 0.032 (Figure 4c). It is clearly porous at φ_v_ = 0.128 and 0.064, although this is less pronounced at φ_v_ = 0.032. It differs therefore from the dense zone formed adjacent to the cold source of a liquid nitrogen-charged cold-finger [16]. With vacuum-induced surface freezing the rate of advancement of the freezing front [18] is evidently sufficient low to allow horizontal accumulation of the nanoparticles between the aligned lamellar ice crystals being formed, but not high enough to prevent some of the nanoparticles being pushed downwards. These then accumulate as a layer at the base at the end of the freezing process. Recall that with vacuum-induced surface freezing the freezing front moves from top to bottom and not from bottom to top as in regular freeze casting with a liquid nitrogen-charged cold-finger [16]. This explains why this layer has low porosity and its height is a direct function of slurry concentration. The formation of a close-packed, cohesive layer of coagulated, rejected nanoparticles has been observed in the unfrozen region ahead of the freezing front at low freezing rates of alumina slurries (≤3 µm/s) [26,27]. This was associated with ice segregation as crack-like ice-lenses. Yet the dense base-layers observed in Figure 4a–c differ from this behavior in that they are formed at higher freezing rates (estimated to be up to 30 µm/s [18]). Additionally, the region directly adjacent to the dense base-layer, i.e., that formed from the segregated ice crystals, is the aligned lamellar region, where no indication of an ice-lens morphology is observed. Note that the effects of gravity in forming this dense base layer will be weak. Stokes’ Law for a non-interacting sphere falling through a fluid gives a sedimentation rate of 310 nm/s for the alumina nanoparticles through water at 10 °C, which is only one-hundredth part of the estimated initial rate of advancement of the freezing front during vacuum-induced surface freezing [18].

**Figure 5:**
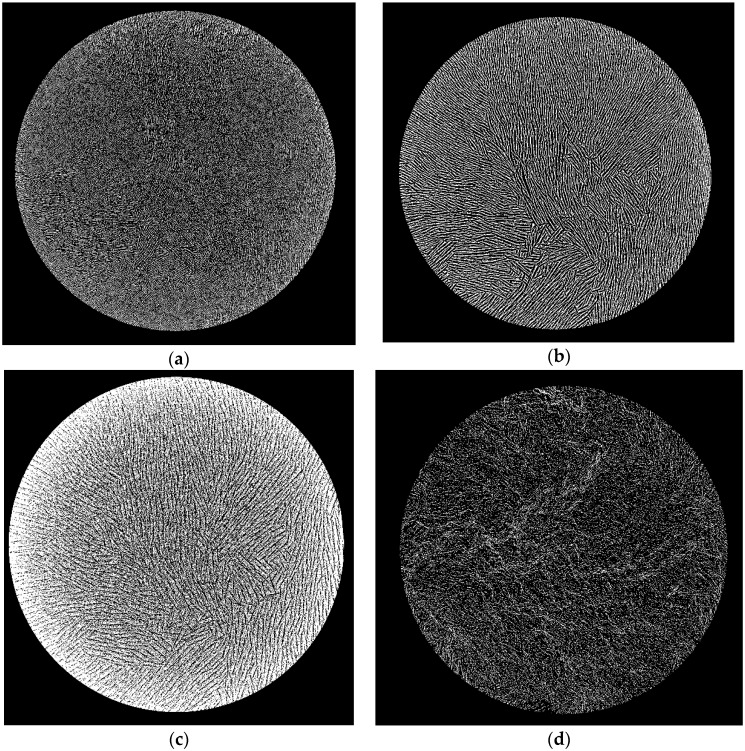
X-ray micro-computed tomography images of cross-sections through monoliths at different heights, h (cf. Figure 1a). (**a**) φ_v_ = 0.128, h = 0.90; (**b**) φ_v_ = 0.128, h = 0.5; (**c**) φ_v_ = 0.128, h = 0.18; (**d**) φ_v_ = 0.032, h = 0.9.

**Figure 6:**
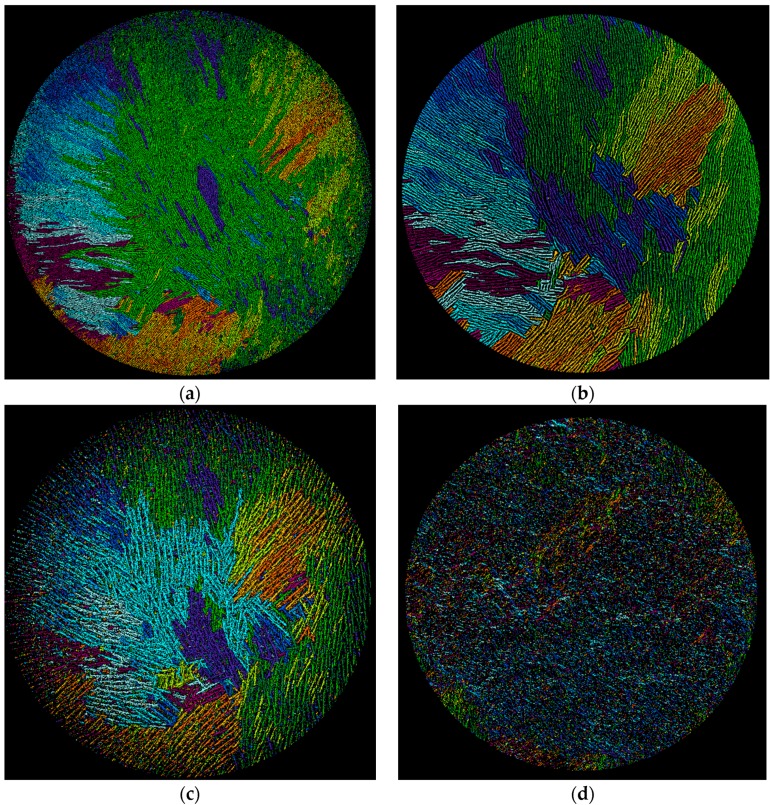
Image-analyzed X-ray micro-computed tomographs of images in Figure 5a–d to reveal orientation of domains at different heights, h, within monolith, h (cf. Figure 1a and Figure 5). (**a**) φ_v_ = 0.128, h = 0.90, pores image; (**b**) φ_v_ = 0.128, h = 0.5, lamellae image; (**c**) φ_v_ = 0.128, h = 0.18, pores image; (**d**) φ_v_ = 0.032, h = 0.9, lamellae image.

X-ray micro-computed tomography (µCT) gives sinograms that visualize directly the three-dimensional pore network within the sintered monoliths. The vertical arrangement of the lamellae and pores is revealed by taking two-dimensional cross-sections through each monolith at different vertical heights, h, of approximately 0.9, 0.5 and 0.1, each at ±50 slices (approximately 2200 slices each of 8.9 µm thickness were resolved over the total monolith height, h = 1, of approximately two centimeters). The positions of h within the φ_v_ = 0.128 membrane are marked in Figure 1a and the corresponding cross section images given in Figure 5a–c. At h = 0.9, i.e., at 90% of total height, Figure 5a shows the pattern of dark pores that are separated by the lighter-shaded lamellae of the sintered alumina. It is immediately obvious that the pores and lamellae are not oriented uniformly over the cross-section. In the left-hand hemisphere the pores and lamellae in the periphery are oriented radially. In the right-hand hemisphere many pores and lamellae run across the periphery as parallel chords. On moving away from the periphery towards the center the lamellae and pores now exist as individual domains, where each domain comprises a group of parallel lamellae/pores. The numerous domains are randomly oriented to one another. The existence of such domains or “colonies” has been observed before in regularly freeze-casted ceramics at “high” freezing rates (i.e., 20–30 µm/s [28]) and have the same intra-domain alignment of pores [28,29] but with no long-range order [30]. Recall that the estimated freezing rate with vacuum-induced surface freezing is 30 µm/s [18]. This non-uniform orientation over the cross-section explains the appearance of the lamellae visualized by SEM in Figure 1a. When viewed longitudinally, the monolith of Figure 5a will show lamellae sectioned at different angles because of the different orientations of the lamellae/pores in the domains. A similar pattern of lamellae/pore structure is seen deeper in the monolith at h = 0.5 (Figure 5b) with radial and chord orientation in the periphery and random domains in the center. It is, however, evident that both the lamellae and pores are larger at h = 0.5 than at h = 0.9. Despite this, the locations and dimensions of the orientation domains are fairly constant in the two cross-sections. This is shown in the image-analyzed pictures [31] in Figure 6a,b, where the domains of different orientation are colored differently. The orientation domains are fairly constant through the sample between h = 0.9 (Figure 6a) and h = 0.5 (Figure 6b). Note that Figure 6a shows the pores colored and Figure 6b the lamellae to demonstrate the parallel runs of both through the height of the monolith. This means that the lamellae and pores in their domains run more-or-less uniformly vertical and in parallel through the monolith between h = 0.90 and 0.5. This vertical alignment is expected for the case of a predominantly horizontal temperature gradient through the sample during vacuum-induced freezing, as discussed above. Moving further down to h = 0.10 (Figure 5c) we arrive in the base region of the monolith. An aligned porous structure has still been formed, but the light-shaded lamellae are much thicker than higher in the monolith. This is precisely the dense but still aligned porous appearance seen already in the SEM image (cf. Figure 4a) and attributed to accumulation of the nanoparticles as a layer preceding the advancing freezing front with vacuum-induced surface freezing. This occurs despite the apparently “high” freezing rate [18] and absence of any ice-lenses reported before [27]. The orientation domains detected at h = 0.1 (Figure 6c) are also fairly close to those seen the higher sections. This means that the lamellae and pores in their domains run more-or-less uniformly through the monolith from h = 0.9 to h = 0.1 despite the more dense but still porous base layer.

The cross-section images for φ_v_ = 0.064 and 0.032 illustrate the same pattern of domains of lamellae and pore orientation within the lamellar and base layers depending on height as seen at φ_v_ = 0.128 and are therefore not shown here. The singular difference is that the lamellae size appears the same at all heights within each monolith, but does vary with slurry concentration. Furthermore, the sub-surface zone at h = 0.9 shows a disordered structure (Figure 5d for the example of φ_v_ = 0.032), which comes from the granular, cellular appearance in the sub-surface zone observed in the SEMs (cf. Figure 2b,c). This lack of alignment is also recognizable in the image-analyzed picture at h = 0.9 shown in Figure 6d. Taken together, the µ-CT cross-sections through the monoliths prepared by vacuum-induced surface freezing illustrate a very similar, more-or-less uniform vertical and parallel pore structure in the lamellar region to that which is well known with regular freeze casting. The observed existence of domains suggests that vacuum-induced surface freezing has a “high” freezing rate [28] that lies in the range produced with a liquid nitrogen-driven cold-finger, although this has yet to be determined accurately.

**Figure 7:**
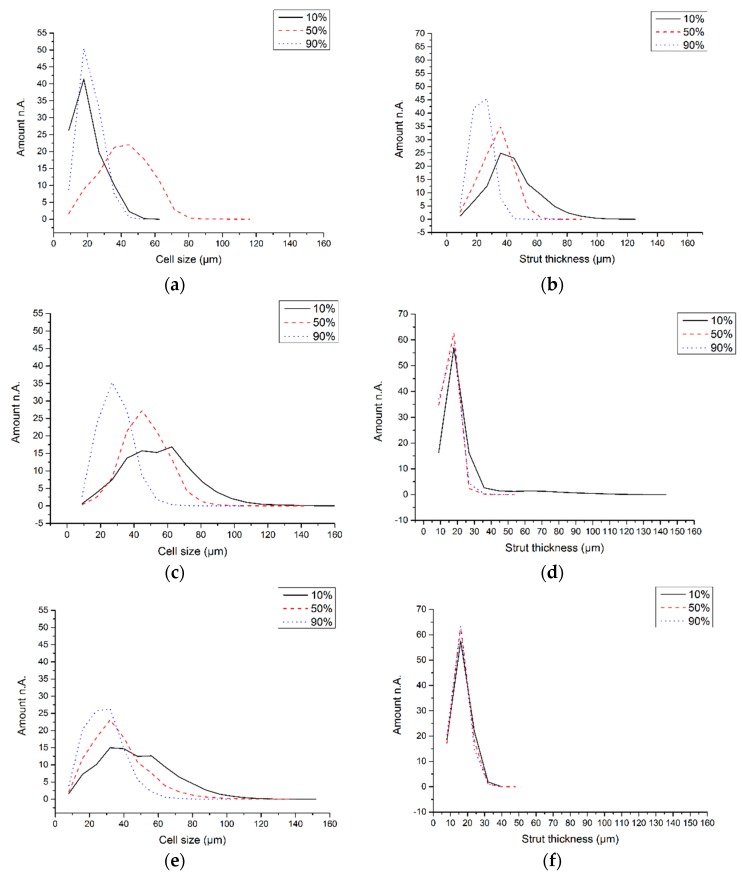
Calculated dimensions at different depths, h, in monoliths prepared at various slurry concentrations, φ_v_. (**a**) φ_v_ = 0.128 Cell size; (**b**) φ_v_ = 0.128 Strut size; (**c**) φ_v_ = 0.064 Cell size; (**d**) φ_v_ = 0.064 Strut size; (**e**) φ_v_ = 0.032 Cell size; (**f**) φ_v_ = 0.032 Strut size.

The cell (pore) diameter and the strut (lamella) thickness were determined from the µCT cross-sections [32]. With φ_v_ = 0.064 and 0.032 the cell size shows a consistent increase moving down from h = 0.9 to 0.1 (Figure 7c,e, respectively), although this is not seen with the most concentrated slurry, φ_v_ = 0.128 (Figure 7a). A widening of the pores with greater depth agrees with measurements of increased wavelength, λ, with larger distance from a liquid nitrogen-driven cold finger source [24]. The cause is a declining rate of advancement of the freezing front as it moves away from the cold source. This was not measured in the current work. Yet, the Stefan equation predicts that the thickness of a growing ice layer at constant cold source temperature increases with the square root of time [33], i.e., the rate of advancement of the ice front must decrease continually with time. Indeed, a low freezing rate of an alumina slurry in a liquid nitrogen-driven cold-finger increased both pore and lamella thickness and hence also λ compared with a higher freezing rate [21]. If the rate of advancement of the freezing away from the frozen surface (=cold source) obeys the Stefan equation, then the result would be the formation of wider aligned ice crystals as freezing rate slows down as it proceeds from top to bottom.

With φ_v_ = 0.064 and 0.032 the strut size is constant when moving down from h = 0.9 to 0.1 (Figure 7d,f, respectively). Again, the φ_v_ = 0.128 slurry departs from this behavior showing an increase with lower height (Figure 6b). This means that λ increases with depth at all slurry concentrations; at φ_v_ = 0.064 and 0.032 this comes from change in pore size and at φ_v_ = 0.128 is a result of larger strut thickness. Preiss et al. [24] found similar behavior with regularly freeze-cast ceramics, where with increasing distance from the cold finger a larger λ was accompanied by an unchanged strut thickness. These authors also reported that reducing the solid content of the ceramic dispersion made the struts thinner and reduced the λ. This same behavior is seen in Figure 7b,d,f across all three slurry concentrations. The changes in pore and lamellae size within a monolith and the dependence on slurry concentration observed with vacuum-induced surface freezing are therefore the same as those seen with regular freeze casting.

Taken together the results of this work show that vacuum-induced surface freezing produces directional freezing and a porous structure that is highly similar to that seen with regular freeze casting using a liquid nitrogen-driven cold finger.

## 3. Materials and Methods

### 3.1. Materials

Aluminum oxide powder, Al_2_O_3_ CT 3000 SG, was used as received from Almatis (Ludwigshafen, Germany). It has a specific surface area (BET) of 7.8 m^2^/g and an arithmetic mean particle diameter of 500 nm. Polyvinyl alcohol (PVA; Mowiol 4–88; MWt 31,000; Aldrich, Munich, Germany), ammonium polymethacylate anionic dispersant (Darvan CN, RT Vanderbilt Co., Norwalk, CT, USA), and Tego Airex 901 W defoaming agent (Evonik, Frankfurt, Germany) were also all used as received. Water was double-distilled from an all-glass apparatus.

### 3.2. Preparation of Sintered Alumina Monoliths

Slurries were prepared by dispersing the Al_2_O_3_ powder in water to weight fractions, φ_w_, of 0.5, 0.25, and 0.125 (=50% *w*/*w*, 25% *w*/*w* or 12.5% *w*/*w*). For a density of alumina, ρ, of 3.9 kg·m^−3^ these are equivalent to volume fractions, φ_v_, of 0.128, 0.064 and 0.032, respectively. The total slurry content also contained 0.7% *w*/*w* PVA/DARVAN as a binding agent and 0.175% *w*/*w* TEGO Airex to prevent excessive foaming under vacuum. Each slurry was stirred for 24 h at room temperature before being transferred as 3 mL portions into 5 mL glass vials (Carl Roth, Karlsruhe, Germany). The vials were then placed on a precooled shelf at shelf temperature, T_shelf_, of +10 °C in a Virtis Genesis 25 EL lyophilizer and left for approximately 35 min to equilibrate. The chamber pressure, P_cham_, was then reduced to 150 mTorr (≈0.2 mbar). Incipient surface freezing of the slurry was observed when P_cham_ reached approximately 450 mTorr (≈0.6 mbar), just as reported previously with aqueous carbohydrate solutions [17]. P_cham_ was held constant at 150 mTorr, which resulted in a visible unidirectional ice crystal growth through the dispersion from top to bottom. Once the unidirectional crystallization had reached the vial base (after some 10 min) and was deemed to be complete, the vacuum was released and T_shelf_ was reduced rapidly (10 °C/min) to −18 °C to prevent ice melting. This was left to equilibrate for 1 h before P_cham_ was reduced to 30 mTorr (=0.04 mbar) to induce primary drying. This was left to run until the difference in P_cham_ between Pirani and capacitance manometers was ≤3 mTorr. The secondary drying phase was then started by increasing T_shelf_ at 0.12 °C/min over 6 h and keeping P_cham_ at 30 mTorr. At the end of the lyophilization cycle the chamber was flooded with dry nitrogen gas and the vials removed for further treatment. Each green body was removed carefully from its glass vial and then sintered in an air furnace (Hochtemperaturofen HT16/16, Nabertherm GmbH, Lilienthal, Germany) for 2 h. The furnace temperature was first increased to 400 °C at 1 °C/min to burn off the PVA and produce debinding of the colloidal alumina particles. Further heating to the sintering temperature of 1700 °C was then done at 300 °C/h. After sintering was complete, the cooling rate was 300 °C/h down to room temperature.

### 3.3. Scanning Electron Microscopy (SEM)

The intact monoliths were examined on an Amray 1810 T SEM (Amray Inc., Bedford, MA, USA) at 20 kV. Each monolith was fixed on an Al stub and Au sputtered before examination.

### 3.4. X-ray Micro-Computed Tomography (µCT)

The monoliths were examined intact on a Skyscan 1171 MCT (Skyscan, Kontich, Belgium) fitted to an 11 MPix detector. The X-ray tube operated at 80 kV and 100 µA using an additional Al 0.5 mm filter to cut off low-energy X-rays. Each sample was scanned under the following conditions: rotation step = 0.25° over 360°; exposure time = 1765 ms per slice; random movement = 10; giving a resolution of 4.00–4.47 µm per pixel. The raw data sinograms were reconstructed with the tomographic software Nrecon Client & Server 1.6.9 with GPU support (Skyscan, Kontich, Belgium). This calculated the two-dimensional cross-sections of the sample after adjusting grey value levels. The three-dimensional images were then generated using the imaging software Amira 5.5.0 (Visual Imaging, Berlin, Germany) after labeling with a global threshold and using a 26-side growing algorithm on all slices. The 2D cross-section images were binarized using a common threshold and separated into individual objects using CT-Analyser Software 1.16.1 (Bruker, Leuven, Belgium). Orientation was calculated for the major axis of each individual object for the upper hemisphere (0°–180°) and using a random color scale for degree of orientation [31]. The pore structure was quantified as described before [32] by determining the cell diameter and the strut thickness.

## 4. Conclusions

(i) Vacuum-induced surface freezing can be used to produce aligned ice crystal growth through alumina dispersions. It leads to zones of structure within the resulting sintered monolith that are highly similar to those found before with freeze casting using a liquid nitrogen-driven cold source.
(ii) A more-or-less dense surface and a cellular sub-surface region are observed as well as a broad region of aligned lamellae and pores. The latter extend through most of the depth of the monolith, even at a volume fraction of dispersed phase as low as 0.032.
(iii) A denser base layer is caused by accumulation of rejected nanoparticles preceding the freezing front. This differs from similar behavior reported in the literature for high freezing rates and formation of ice lenses, which were not seen here.
(iv) X-ray micro-computed tomography reveals a uniformly directional and aligned pore structure vertically through the lamellar region of the monolith. The pores and lamellae are not, however, oriented uniformly horizontally over the cross-section. Those close to the periphery are oriented radially or as chords, while the center region contains domains of parallel pores/lamellae. The domains are randomly oriented to one another and are the same patterns reported in the literature for regular freeze casting.
(vi) Vacuum-induced surface freezing is convenient and easy to perform, although practise is needed to control the chamber pressure gradient to avoid boiling of the sample under high vacuum. Control of the process requires further investigation of how the temperature gradient across the sample, and hence the freezing rate, can be adjusted by varying the conditions of the chamber pressure and shelf temperature.
